# Effects of Winter Flounder Antifreeze Protein on the Growth of Ice Particles in an Ice Slurry Flow in Mini-Channels

**DOI:** 10.3390/biom9020070

**Published:** 2019-02-18

**Authors:** Yuki Takeshita, Tomonori Waku, Peter W. Wilson, Yoshimichi Hagiwara

**Affiliations:** 1 Division of Mechanophysics, Graduate School of Science and Technology, Kyoto Institute of Technology, Matsugasaki, Sakyo-ku, Kyoto 606-8585, Japan; yuki.takeshita333@gmail.com; 2 Faculty of Molecular Chemistry and Engineering, Kyoto Institute of Technology, Matsugasaki, Sakyo-ku, Kyoto 606-8585, Japan; waku1214@kit.ac.jp; 3 School of Environment, Science and Engineering, Southern Cross University, Military Road, Lismore 2480, NSW, Australia; peter.w.wilson@utas.edu.au; 4 Faculty of Mechanical Engineering, Kyoto Institute of Technology, Matsugasaki, Sakyo-ku, Kyoto 606-8585, Japan

**Keywords:** winter flounder antifreeze protein, solution flow, mini-channel, ice growth, preheating, aggregates, ultrafiltration

## Abstract

The control of ice growth in ice slurry is important for many fields, including (a) the cooling of the brain during cardiac arrest, (b) the storage and transportation of fresh fish and fruits, and (c) the development of distributed air-conditioning systems. One of the promising methods for the control is to use a substance such as antifreeze protein. We have observed and report here growth states of ice particles in both quiescent and flowing aqueous solutions of winter flounder antifreeze proteins in mini-channels with a microscope. We also measured ice growth rates. Our aim was to improve the levels of ice growth inhibition by subjecting the antifreeze protein solution both to preheating and to concentrating by ultrafiltration. We have found that the ice growth inhibition by the antifreeze protein decreased in flowing solutions compared with that in quiescent solutions. In addition, unlike unidirectional freezing experiments, the preheating of the antifreeze protein solution reduced the ice growth inhibition properties. This is because the direction of flow, containing HPLC6 and its aggregates, to the ice particle surfaces can change as the ice particle grows, and thus the probability of interaction between HPLC6 and ice surfaces does not increase. In contrast to this, ultrafiltration after preheating the solution improved the ice growth inhibition. This may be due to the interaction between ice surfaces and many aggregates in the concentrates.

## 1. Introduction

The inhibition of ice growth has been the focus of significant recent research. This inhibition is important for many fields, including (a) cryotherapy [1], (b) the use of ice slurries (the mixtures of ice and water) to cool the brain during cardiac arrest, (c) the preservation of organs for transplantation in hospitals [2], (d) the improvement of storage and transportation of fresh foods by using ice slurries, and (e) the development of distributed air-conditioning systems by using ice slurries. In these cases, there exists the need to control ice growth, at least in the cases of blood plasma and ice slurries.

One of the most promising methods for controlling the growth of ice is to use a substance which has the specific purpose of controlling ice growth. Antifreeze proteins (AFPs) or antifreeze glycoproteins (AFGPs) are appropriate substances for controlling ice growth, because they lower the temperature at which a seed crystal grows but do not alter the temperature at which the crystal is stable during melting at very low cooling rates. Furthermore, an appropriate concentration of AF(G)Ps is much lower than that of other solutes [3]. This minimizes the effect of AF(G)Ps on osmotic pressure.

Among various AF(G)Ps discovered to date, HPLC6, the major fraction of winter flounder AFP, has been widely investigated. Measurements of ice crystal growth and ice crystal morphology in solutions of this protein are classified into the following two types according to the cooling rates: (i) at a very low cooling rate (≤−1 °C/min) and (ii) at a low cooling rate (>−1.5 °C/min). In type (i) measurements, an AFP solution of 0.01 mm^3^ in oil or liquid paraffin in osmometers are typically frozen at −40 °C, and gradually heated until only a single crystal of approximately 7 μm in diameter is obtained [3,4]. The solution with the tiny crystal is maintained at this temperature for 1 min to allow the crystal to stabilize, and this temperature is defined as the ‘melting point’ (mp), although in some cases the mp is taken to be the temperature at which the crystal finally disappears. The solution with the crystal is then gradually cooled at −1.0 °C/min. Chao et al. [5] considered the ‘freezing point’ to be the temperature at which the ice growth velocity exceeded 0.2 μm/s. It can be considered from this low ice-growth velocity that thermodynamically quasi-equilibrium conditions are held. In the case of type (ii) measurements, there are several other experimental methods that can help to elucidate the activities of AF(G)P: Wilson et al. [6] developed an automated lag-time apparatus where a 200 mm^3^ sample solution of winter flounder AFP was held in a glass tube, and they measured the nucleation rate in the tube. Coger et al. [7], Furukawa et al. [8], Butler [9] Hagiwara and Yamamoto [10], Hagiwara and Aomatsu [11] and Miyamoto et al. [12] carried out experiments on the unidirectional freezing of AF(G)P solutions in narrow spaces between two glass plates. They measured the velocities and morphologies of the ice/solution interfaces, the concentration of solutes, temperature distribution and the dimension of AFP aggregates. The ice growth velocity was in the range of 0–89 μm/s. Serrated interfaces were produced by not only the adsorption of AF(G)P but also the approach of AFP aggregates or high concentration region of solute to the interfaces. The concentration distributions of AFP and ions in the mixed solution were changed by the other solute, which is a possible reason for the synergistic effect of solutes.

Although these findings are valid, it has not yet been confirmed whether they hold in the situation where the AFP solution flows along with ice crystals. Furthermore, experimental results for such flows are limited. Grandum et al. [13] showed that the pressure drop for the ice slurry flow was higher than that for water flow in a pipe of 6 mm in inner diameter. They also observed the growth of seed crystals in the direction of *c*-axis in the quiescent solution and the crystals being transported in the core region of flow. Onishi et al. [14] found that HPLC6 inhibited the aggregation of ice particles in ice slurry flow in a mini-channel.

With this in mind, in the present study, we measure the growth of ice particles in the flow of HPLC6 solution in mini-channels. We discuss the effects of preheating or ultrafiltration of the solution on the ice-particle growth.

## 2. Materials and Methods

### 2.1. Materals

The sequence of amino-acid residues for HPLC6 is as follows: DTASDAAAAAALTAANAKAAAELTAANAAAAAAATAR (A: Alanine, D: Aspartic acid, E: Glutamic acid, K: Lysine, L: Leucine, N: Asparagine, R: Arginine, S: Serine, T: Threonine). It consists of 37 amino acid residues and has a molecular weight of 3242 Da. The secondary structure of HPLC6 is an α-helix. Four threonine residues are positioned at nearly identical distances on one line parallel to the helical axis. The distance between the oxygen atoms on the pyramidal faces of ice crystals, which were observed in the supercooled solution of HPLC6, is nearly identical to the distance between the threonine residues. It had thus been hypothesized that the hydrogen atoms of the threonine residues were bonded permanently to the oxygen atoms on the pyramidal faces in the ice crystal and that the water molecules were prevented from bonding to the ice surface by the Kelvin (or Gibbs–Thomson) effect [5]. On the other hand, discussion was conducted on the alanine-rich surfaces of HPLC6 in the ice-growth inhibition mechanism [15,16,17].

We purchased the synthetic polypeptide from GenScript Inc. (Taito, Tokyo, Japan).

### 2.2. Preheating

The HPLC6 solution was preheated at a constant temperature for a predetermined time and cooled in the temperature-controlled room before the measurements were carried out. A polypropylene bottle containing the sample liquid was installed in a drying chamber (As One Co., Ltd., Osaka, Japan, ETTAS ONW-450S). The temperature was set to 80 °C and the duration was one hour. The HPLC6 concentration was 0.5 mg/mL. The preheating condition was determined based on our previous study on the inhibition of unidirectional freezing of HPLC6 solution [12].

### 2.3. Ultrafiltration

The preheated HPLC6 solution was filtered to increase the solution concentration. We used a centrifuge (Koki Holdings Co., Ltd., Tokyo, Japan, Himac CF15D2) and ultrafiltration membranes. The molecular weight cut-offs of membranes were 10^4^ and 10^5^. The centrifugal force was 14,000× *g*.

### 2.4. Production of Ice Slurry

**Figure 1:**
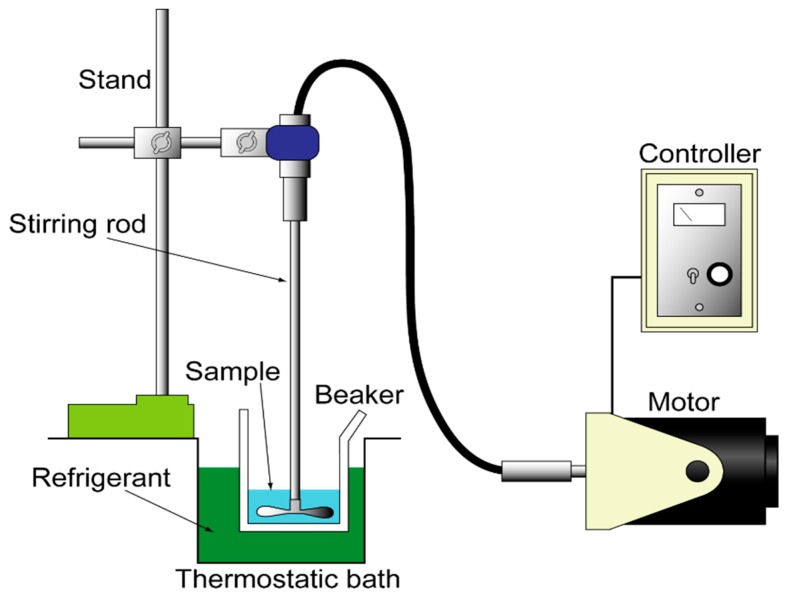
Apparatus for producing ice slurry.

Figure 1 shows the apparatus for producing ice slurry. Saline solution of 100 mL was contained in a beaker made of polymethylpentene. The beaker was installed in the constant-temperature liquid bath (Yamato Scientific Co., Ltd., Tokyo, Japan, BB301). The bath was filled with ethylene glycol as a coolant. The free surface of the coolant was set higher than that of the saline solution so that the solution was cooled from surroundings.

First, the temperature of the solution was maintained at −3 °C. Secondly, seed crystals of ice were added to the supercooled solution. The solution with ice particles was stirred with an agitator (As One Co., Ltd., Osaka, Japan, K-2RFN) for one hour. The temperature of the mixture was maintained at −1.2 °C.

### 2.5. Apparatus

The apparatus consisted of an inverted biological microscope (Nikon Instech Co., Ltd., Tokyo, JapanNikon, C2+), a monochrome charge-coupled device (CCD) video camera (Hamamatsu Photonics K. K., Hamamatsu, Japan, ORCA-ER), a bench-top cooling section and a syringe pump (Harvard Apparatus, Holliston, Massachusetts, USA, Model 11 Elite). The apparatus was placed in a temperature-controlled room maintained at 2 °C.

### 2.6. Mini-Channels

**Figure 2:**
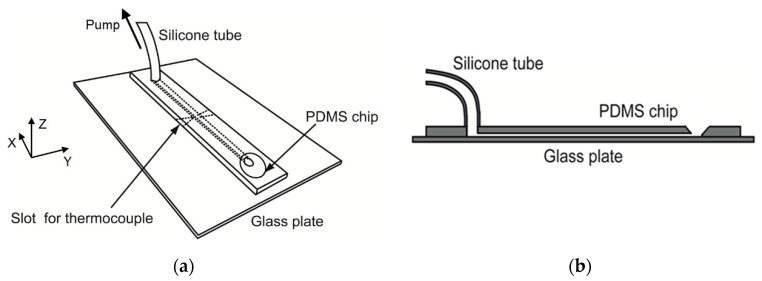
Mini-channel: (**a**) Bird’s-eye view; (**b**) Cross section.

Figure 2 shows the mini-channel. The channel was made of a grooved polydimethylsiloxane (PDMS) plate of thickness 1 mm, width 10 mm and length 70 mm, and a flat glass plate of thickness 1 mm, width 52 mm and length 76 mm. These plates were glued to each other. The following two mini-channels were used: tThe dimensions of the mini-channel 1 were depth 1.0 mm, width 2.0 mm and length 50 mm, whereas the dimensions of the mini-channel 2 were depth 0.70 mm, width 1.0 mm and length 50 mm. These channels are models for blood vessels in the applications of (a)–(d) mentioned in the introduction. We arranged the coordinates as follows: The *X*-axis was in the slurry-flow direction, the *Y*-axis was in the transverse direction, and the *Z*-axis was in the vertical (viewing) direction. The origin of the coordinates was located at the corner of the channel bottom surface.

A silicone tube of inner diameter 2 mm and outer diameter 3 mm was attached to the channel exit. The tube was connected to the syringe pump to draw ice slurry from the channel. A syringe, which consisted of a polypropylene cylinder and a polyethylene piston, was used. The volume of the syringe was 1 mL. The flow rate of slurry was set at 0, 20 and 40 μL/min.

The ice slurry with or without HPLC6 was introduced into the mini-channels from the cone-shaped entrance. The ice slurry was mixed manually with a small stirrer at the entrance.

### 2.7. Cooling of Channels

We adopted the following two types of channel cooling: The ambient air temperature was 0.5 °C lowered to subzero degrees (type A), and copper plates surrounding the channel were cooled by a thermoelectric cooler (i.e., Peltier cooler) (type B). In the case of type B cooling, the channel axis was in parallel with the axis of conductive heat transfer of the copper plates. The flow direction was opposite to the conduction. The copper plates and the thermoelectric cooler were adhered with heat conduction grease. The copper plates had slits of width 2 mm to illuminate and observe the channel. The temperature inside the cooler was set at −2.0 °C.

### 2.8. Image-Capturing Condition

The image-capturing condition is shown in Table 1. In the case of quiescent ice slurry containing HPLC6, we used the mini-channel 1 under the type A cooling condition. The concentration of HPLC6 was varied as 0, 0.125, 0.25 and 0.50 mg/mL. The measured temperature around the observation area was −1.2 °C.

In the case of ice slurry flow containing HPLC6, we adopted the identical mini channel, the cooling condition and the image-capturing condition. The concentration of HPLC6 was 0.25 mg/mL. The flow rate was 40 μL/min. The observation area was moved downstream so that the images of identical ice particles could be captured. The measured temperature around the observation area was −1.4 °C.

In the case of flow of the mixture of ice slurry with the preheated solution of HPLC6, we used the mini-channel 2 under the type B cooling condition. We adopted the identical image-capturing condition. The concentration of HPLC6 was 0.25 or 0.50 mg/mL. The concentration of sodium chloride was 0.45 wt%. The measured temperature around the observation area was 1.0 °C.

In the case of flow of the mixture of ice slurry with the ultra-filtrated solution of preheated HPLC6, the mini-channel and the cooling condition were identical with those used in the case of flow of the mixture of ice slurry with the preheated solution of HPLC6. We changed the volume of HPLC6 solution for its mixing with the ice slurry and the molecular-weight limit of ultrafiltration.

## 3. Results and Discussion

### 3.1. In the Case of Quiescent Ice Slurry

#### 3.1.1. Ice Particle Shapes

**Figure 3:**
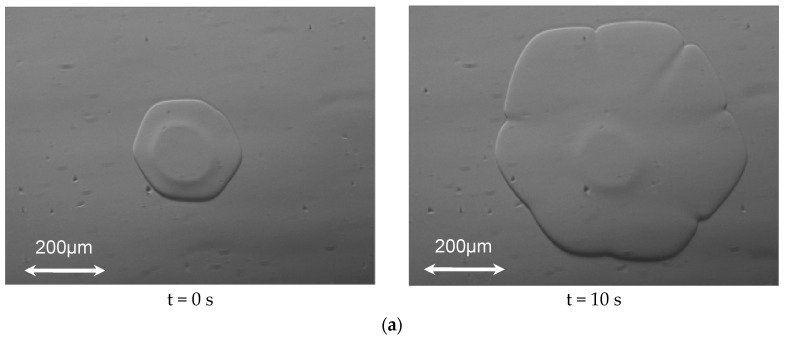

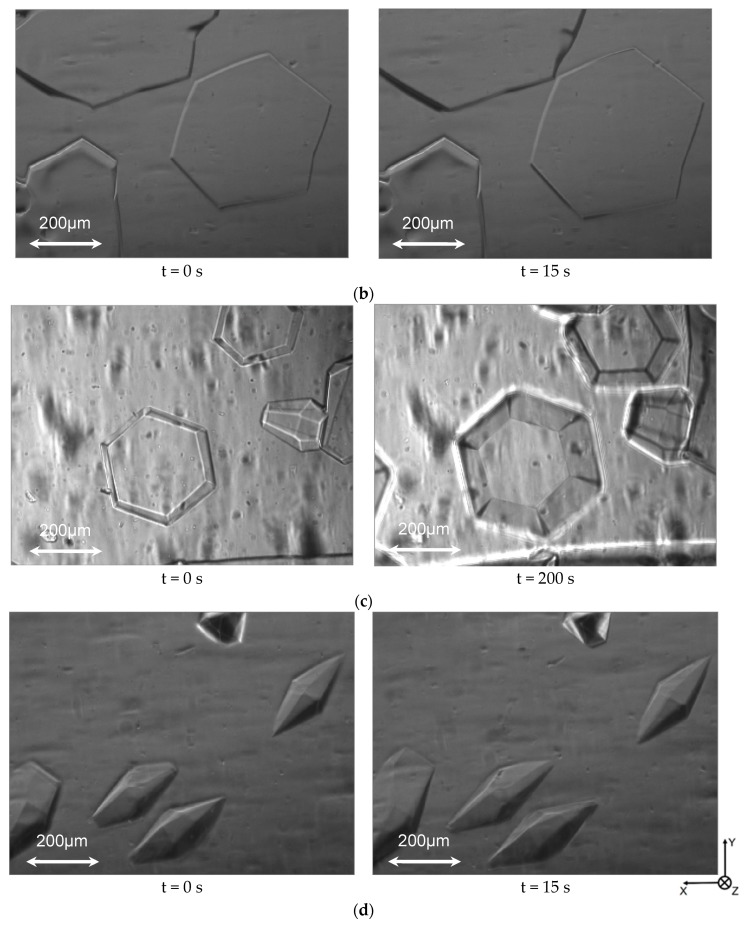
Shapes of typical ice particles in the quiescent ice slurry. (**a**) In the case of c = 0 mg/mL; (**b**) In the case of c = 0.125 mg/mL; (**c**) In the case of c = 0.25 mg/mL; (**d**) In the case of c = 0.50 mg/mL.

Figure 3 shows typical ice particles. In the case of quiescent ice slurry without HPLC6, the ice particles had shapes of elliptical plates, when the image capturing started. Some elliptical plates grew uniformly, whereas the other plates grew non-uniformly (see Figure 3a). The latter plates formed dendrite crystals eventually. The ice grew along the *a*-axis because the growth rate along the *a*-axis is more than 100 times higher than the growth rate along the *c*-axis [18]. The ice plates were located near the inner surface of the upper wall of the mini-channel due to buoyancy.

In the case of quiescent ice slurry with HPLC6 whose concentration was 0.125 mg/mL, the ice particles became plates with the shape of a hexagon when the image-capturing started (see Figure 3b). These plates grew in the normal direction to the edge surfaces of the hexagonal shape, i.e., in the *a*-axis direction.

In the case where the HPLC6 concentration was 0.25 mg/mL, as shown in Figure 3c, the edge surfaces of large hexagonal plates are found to be wider than the edge surfaces in Figure 3b. This clearly shows that the edge surfaces had already leaned to the *z*-axis (i.e., the *c*-axes of the ice particles) before the image-capturing started. A small ice particle whose *c*-axis is parallel to the images is also seen in this figure. After 200 s, the hexagonal shapes became larger, and the edge surfaces of large hexagonal plates became much wider than before. That is, ice particles grew in the *a*-axis and *c*-axis directions, and the edge surfaces inclined further. Consequently, the large plates seemed to become truncated hexagonal pyramids. The small ice particle became a truncated hexagonal bi-pyramid. Note that these truncated pyramids became pyramids eventually.

In the case where the HPLC6 concentration was highest, the ice particles had already had shapes of hexagonal bi-pyramid before the image-capturing started (see Figure 3d). The hexagonal bi-pyramidal shape is similar to the ice crystal shape commonly observed in nanoliter osmometers, the shape being the result of adsorption of HPLC6 on the ice surfaces. The axes of the bi-pyramidal ice crystals are not parallel but perpendicular to the *z*-axis. This is probably a result of slow flow of ice slurry in the mini-channel to introduce ice particles into the central part of the channel. Thus, it is surmised that the adsorption of HPLC6 on the pyramidal faces of ice particles had already initiated just after the ice slurry and HPLC6 solution were mixed.

#### 3.1.2. Ice Growth Rate

**Figure 4:**
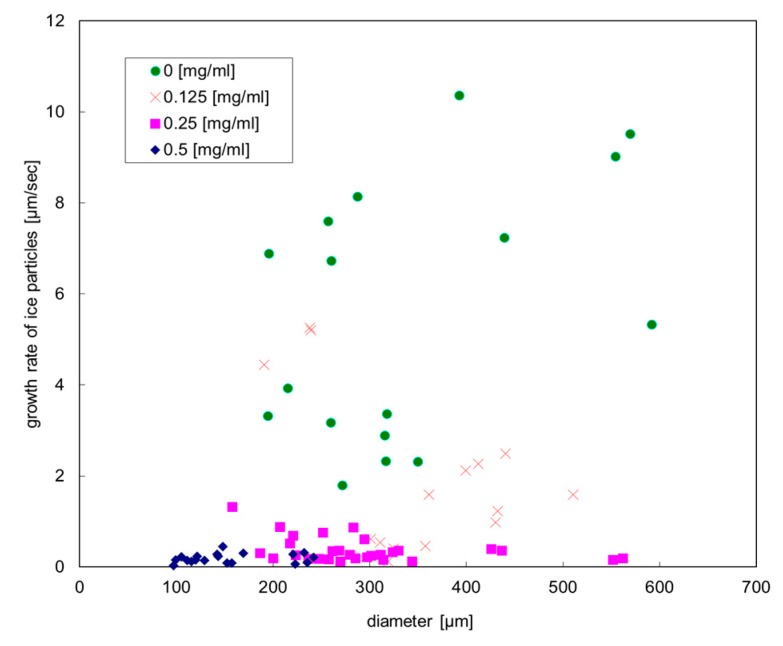
Relationship between the diameters of ice particles and the growth rates of the ice particles.

Figure 4 shows the relationship between the diameters of ice particles and the growth rates of the ice particles which were obtained from the successive images. The horizontal axis shows the diameter of an ice particle in the *a*-axis direction, while the vertical axis shows the ice particle growth rate in the identical direction. (The growth rate in the case of elliptic plates was defined by the increase in the major axis.) It turns out that both the diameter and growth rate of ice particles in the case of the highest concentration of HPLC6 are lower compared with those in the cases of the lower concentrations. In the case without HPLC6, the diameter and the growth rate are scattered in wide ranges. Generally, the scattering of the diameter and growth rate decreased with an increase in the HPLC6 concentration.

Table 2 shows the average value of the growth rate of the ice particles. The growth rate is found to decrease noticeably with an increase in the concentration. The attenuation of ice growth in the *a*-axis direction causes the enhancement of ice growth in the *c*-axis direction. Consequently, the hypothesis in which the pyramidal faces become the slowest surface through the ice crystal growth in osmometers stands even for the ice particles in the mini-channel.

### 3.2. In the Case of Ice Slurry Flow

**Figure 5:**
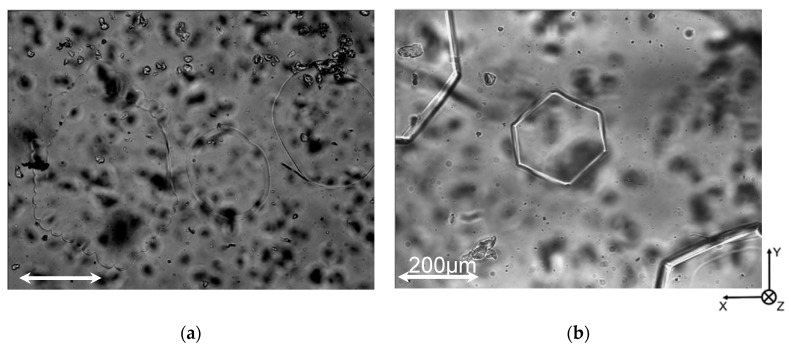
Typical ice particles observed in the flow of ice slurry. (**a**) In the case of c = 0 mg/mL; (**b**) In the case of c = 0.25 mg/mL.

Figure 5 shows typical ice particles observed in the flow of ice slurry. Black dots in the figure are the shadows of dust deposited on the outer surfaces of the channel. In the case of ice slurry flow without HPLC6, ice particle plates had round shapes (see Figure 5a). The shapes are similar to those in the case of quiescent ice slurry shown in Figure 3a. However, the edges of these plates were partially round and partially serrated, which is different from those observed in the case of quiescent ice slurry.

In the case where the HPLC6 concentration was 0.25 mg/mL, as shown in Figure 5b, hexagonal ice plates were seen. The shapes are similar to those in the case of quiescent ice slurry shown in Figure 3c. However, the edge surfaces of these plates were thinner than those seen in Figure 3c. This shows that the edge surfaces are not inclined to the *z*-axis, or to the *c*-axes of the ice particles.

Table 3 compares the average growth rates of ice particles. It is found that the average growth rates in the cases of ice slurry flow are higher than those in the cases of quiescent ice slurry, irrespective of the HPLC6 concentration. This is because the solvent flow carried both HPLC6 and the latent heat away from the surfaces of ice particles. This lowered the interaction between HPLC6 and ice surfaces. Nevertheless, the addition of HPLC6 to the ice slurry flow is effective to control of ice growth in flow.

### 3.3. Effects of Preheating of HPLC6 Solution

In reference [12], the short-time preheating of HPLC6 solution inhibited ice growth and enhanced the supercooled states of the solution. This is because the aggregates of HPLC6, produced by the preheating, contributed to the maintenance of specific configuration of the ice/solution interface and the maintenance of the wide solution regions between ice crystal grains. Thus, we expected similar effects in the case of ice slurry flow.

**Figure 6:**
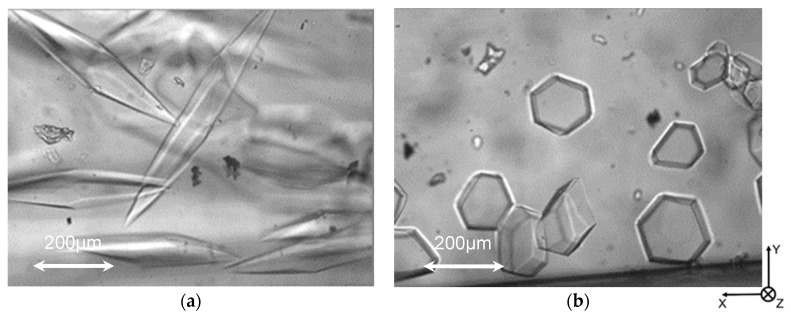
Typical ice particles observed in the flow of ice slurry in the case where the preheated HPLC6 solution was used. (**a**) In the case of unheated HPLC6 solution; (**b**) In the case of preheated HPLC6 solution. Black dots are the shadows of dust deposited on the outer surfaces of the channel.

Figure 6 shows typical ice particles observed in the flow of ice slurry. In the case where the unheated HPLC6 solution was used, ice crystals with the shapes of hexagonal bi-pyramids were observed as shown in Figure 6a. These ice crystals are not similar to those seen in Figure 5b, though the concentration of HPLC6 was identical. This is because only small ice particles could be introduced into the mini-channel 2, since the cross-sectional area of the mini-channel 2 is approximately one-third of the mini-channel 1 used for the flow described in Section 3.2. Furthermore, the temperature in this case is lower than that in the case of slurry flow in Section 3.2.

In the case where the preheated HPLC6 solution was used, on the other hand, ice crystals with the shapes of truncated hexagonal pyramids were observed, as shown in Figure 6b. From the successive images including this image, it was found that these ice crystals grew along the *a*-axis. Table 4 show the average growth rate of ice particles along the *a*-axis. It is found that the average growth rate in the case where the preheated HPLC6 solution was used is higher than that in the case where the unheated HPLC6 solution was used. In addition, the standard deviation in the case where the preheated HPLC6 solution was used is higher than the unheated HPLC6 solution. These facts show that the interaction between HPLC6 and ice particles, such as the adsorption of HPLC6 on ice surfaces, was not strong in the case where the preheated HPLC6 solution was used. This is different from the experimental results for the unidirectional freezing of HPLC6 solution obtained by Miyamoto et al. [12]. The HPLC6 and its aggregates must approach the ice surfaces in the unidirectional freezing, whereas the solvent flow around the ice particle surfaces tends to leave from the adjacent areas of ice surfaces as the ice grows in the ice slurry flow. As a result, the probability of interaction between HPLC6 and ice surfaces does not increase with the ice particle growth. This is the reason for the small effect of the preheating on the ice growth inhibition in the case of ice slurry flow.

### 3.4. Effects of Ultrafiltration of Preheated HPLC6 Solution

To improve the activity of ice growth inhibition in the case where the preheated HPLC6 was used, we carried out ultrafiltration for the solution. To examine the effects of the ultrafiltration, we measured the growth rates of ice particles in the flow of ice slurry in the cases where the filtrate and concentrate were used.

**Figure 7:**
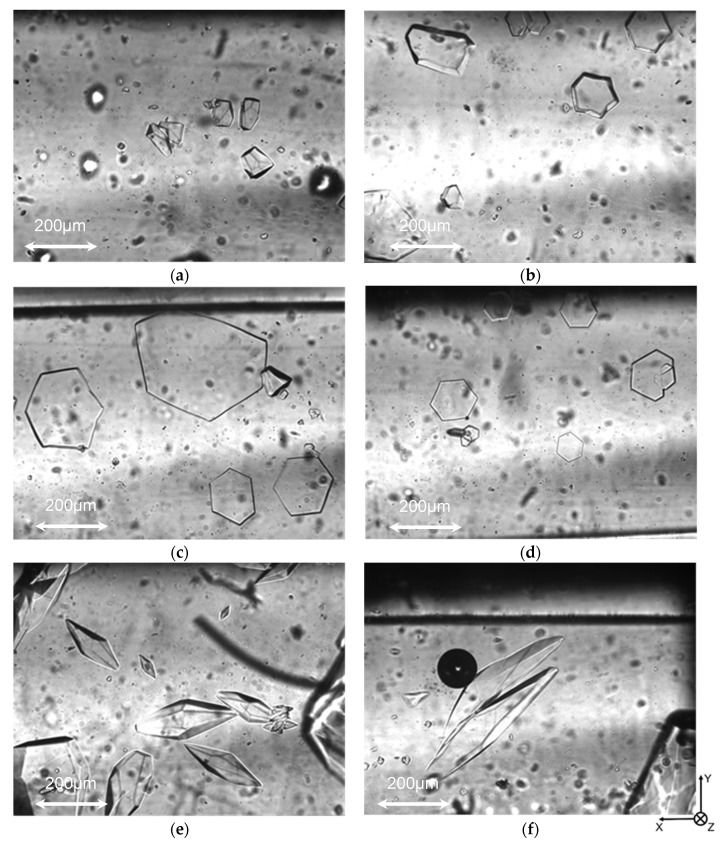
Shapes of typical ice particles in the quiescent ice slurry. (**a**) In the case of filtrate. Molecular weight limit was 10^5^; (**b**) In the case of filtrate. Molecular weight limit was 10^5^; (**c**) In the case of concentrate. Volume was one-tenth. Molecular weight limit was 10^5^; (**d**) In the case of concentrate. Volume was one-tenth. Molecular weight limit was 10^4^; (**e**) In the case of concentrate. Molecular weight limit was 10^5^; (**f**) In the case of concentrate. Molecular weight limit was 10^4^. A black circle in this figure is the shadow of a micro bubble in the channel, which did not affect the motion of ice particles significantly.

Figure 7 shows the images of typical ice particles. In the cases where the filtrate of preheated HPLC6 solution was used, ice crystals with the shapes of truncated hexagonal pyramids were observed, as shown in Figure 7a,b. In the cases where a small amount (one-tenth of the normal volume) of concentrate of preheated HPLC6 solution was used, ice crystals with the shapes of hexagonal plates were observed, as shown in Figure 7c,d. In the cases where the concentrate of preheated HPLC6 solution was used, ice crystals with the shapes of truncated hexagonal pyramids were observed, as shown in Figure 7e,f.

Table 5 shows the average growth rate of ice particles along the *a*-axis. It is found that the average growth rates in the case where the filtrates of preheated HPLC6 solution were used are higher than those in the case where the unheated or preheated HPLC6 solution was used. The growth rate for the filtrate in the case of M_w_ = 10^5^ is lower than that in the case of M_w_ = 10^4^. This is because many aggregates whose molecular weight is higher than 10^4^ passed through the membrane and were included in the filtrate. The average growth rates in the case where the small amount of concentrates of preheated HPLC6 solution were used are higher than those in the case where the unheated or preheated HPLC6 solution was used. In contrast with these, the average growth rates in the cases where the concentrates of preheated HPLC6 solution were used are the same, or lower, than those in the case where the unheated or preheated HPLC6 solution was used. In particular, the use of membrane whose molecular weight limit is 10^4^ is found to be effective. This is because many small aggregates and large aggregates remaining in the concentrate interacted with ice particle surfaces effectively. It can be concluded that the combination of short-time preheating and ultrafiltration is effective for the inhibition of ice growth and enhancement of the supercooled states of the carrier fluid flow in ice slurry.

## 4. Conclusions

We have carried out observations of ice particle growth in both quiescent and flowing aqueous solutions of winter flounder antifreeze proteins in a mini-channel and measured ice growth rates. The main conclusions are as follows:

(1) The ice growth inhibition by the antifreeze protein decreased in flowing solutions compared with that in quiescent solutions. This is because the slurry flow carried HPLC6 and the latent heat away from the surfaces of ice particles locally.
(2) The preheating of the antifreeze protein solution before mixing with the ice slurry reduced the ice growth inhibition properties for the ice slurry flow. As the direction of flow, containing HPLC6 and its aggregates, to the ice particle surfaces can change as the ice particle grows, the probability of interaction between HPLC6 and ice surfaces does not increase with the ice particle growth.
(3) The ultrafiltration after preheating the solution improved the ice growth inhibition. In a specific case, the inhibition was enhanced because many small aggregates and large aggregates remaining in the concentrate could interact with ice particle surfaces effectively.

## Figures and Tables

**Table 1:** Image-capturing condition.

	Quiescent, Flowing	Preheated, Ultra-Filtrated
Mini-channel	1	2
Cooling type	A	B
Magnification	×10	×5
Pixel number	1344 × 1024	
Area size (μm^2^)	867 × 660	1234 × 940
Pixel resolution (μm^2^)	0.645 × 0.645	0.918 × 0.918
Binning	1 × 1	
Depth	12 bit	
Exposure time (s)	0.03	

**Table 2:** Average growth rate of ice particles.

c = 0 mg/ml	c = 0.125 mg/mL	c = 0.25 mg/mL	c = 0.50 mg/mL
5.0 (2.7) μm/s	2.0 (1.7) μm/s	0.30 (0.28) μm/s	0.18 (0.10) μm/s

c is the HPLC6 concentration. The values in the brackets show the standard deviation.

**Table 3:** Comparison of average growth rate of ice particles.

Q = 40 μL/min		Q = 0 μL/min	
c = 0 mg/mL	c = 0.25 mg/mL	c = 0 mg/mL	c = 0.25 mg/mL
13 (8.6) μm/s	4.1 (1.8) μm/s	5.0 (2.5) μm/s	0.30 (0.30) μm/s

Q is the flow rate, and c is the HPLC6 concentration. The values in the brackets show the standard deviation.

**Table 4:** Average growth rate of ice particles in the case where the HPLC6 solution was preheated.

c = 0.25 mg/mL		c = 0.50 mg/mL	
Unheated	Preheated	Unheated	Preheated
2.5 (1.2) μm/s	3.8 (2.4) μm/s	0.35 (0.40) μm/s	0.71 (0.85) μm/s

Q = 20 μL/min. The values in the brackets show the standard deviation.

**Table 5:** Average growth rate of ice particles in the case where the HPLC6 solution was filtered.

Normal Vol.	Normal Vol.	Reduced Vol.	Reduced Vol.	Normal Vol.	Normal Vol.
M_w_ = 10^5^	M_w_ = 10^4^	M_w_ = 10^5^	M_w_ = 10^4^	M_w_ = 10^5^	M_w_ = 10^4^
Filtrate	Filtrate	Concentrate	Concentrate	Concentrate	Concentrate
0.94 (0.60) μm/s	1.25 (0.54) μm/s	1.25 (0.59) μm/s	1.11 (0.63) μm/s	0.35 (0.20) μm/s	0.25 (0.22) μm/s

Q = 20 μL/min. c = 0.50 mg/mL. The values in the brackets show the standard deviation. Note that the average growth rates of ice particles in the cases of unheated and preheated solution were 0.35 μm/s and 0.69 μm/s, respectively. The results for the filtrates containing only small aggregates are the reference results. The results for the reduced volume (one-tenth of the volume) are also the reference results.
